# Alternative Splicing of NOX4 in the Failing Human Heart

**DOI:** 10.3389/fphys.2017.00935

**Published:** 2017-11-22

**Authors:** Zoltán V. Varga, Márton Pipicz, Júlia A. Baán, Tamás Baranyai, Gábor Koncsos, Przemyslaw Leszek, Mariusz Kuśmierczyk, Fátima Sánchez-Cabo, Pablo García-Pavía, Gábor J. Brenner, Zoltán Giricz, Tamás Csont, Luca Mendler, Enrique Lara-Pezzi, Pál Pacher, Péter Ferdinandy

**Affiliations:** ^1^Cardiometabolic Research Group, Department of Pharmacology and Pharmacotherapy, Semmelweis University, Budapest, Hungary; ^2^Laboratory of Cardiovascular Physiology and Tissue Injury, National Institute on Alcohol Abuse and Alcoholism, National Institutes of Health, Bethesda, MD, United States; ^3^Department of Biochemistry, Faculty of Medicine, University of Szeged, Szeged, Hungary; ^4^Department of Heart Failure and Transplantology, Cardinal Stefan Wyszyński Institute of Cardiology, Warszawa, Poland; ^5^Department of Cardiac Surgery and Transplantology, Cardinal Stefan Wyszyński Institute of Cardiology, Warszawa, Poland; ^6^Bioinformatics Unit, Centro Nacional de Investigaciones Cardioavsculares Carlos III, Madrid, Spain; ^7^Heart Failure and Inherited Cardiac Diseases Unit, Department of Cardiology, Hospital Universitario Puerta de Hierro Majadahonda, Madrid, Spain; ^8^Pharmahungary Group, Szeged, Hungary; ^9^Faculty of Medicine, Institute of Biochemistry II, Goethe University, Frankfurt, Germany; ^10^Centro de Investigaciones Cardiovasculares Carlos III, Madrid, Spain

**Keywords:** cardiomyopathy, oxidative stress, cardiac dysfunction, myocardium, aging

## Abstract

Increased oxidative stress is a major contributor to the development and progression of heart failure, however, our knowledge on the role of the distinct NADPH oxidase (NOX) isoenzymes, especially on NOX4 is controversial. Therefore, we aimed to characterize NOX4 expression in human samples from healthy and failing hearts. Explanted human heart samples (left and right ventricular, and septal regions) were obtained from patients suffering from heart failure of ischemic or dilated origin. Control samples were obtained from donor hearts that were not used for transplantation. Deep RNA sequencing of the cardiac transcriptome indicated extensive alternative splicing of the NOX4 gene in heart failure as compared to samples from healthy donor hearts. Long distance PCR analysis with a universal 5′-3′ end primer pair, allowing amplification of different splice variants, confirmed the presence of the splice variants. To assess translation of the alternatively spliced transcripts we determined protein expression of NOX4 by using a specific antibody recognizing a conserved region in all variants. Western blot analysis showed up-regulation of the full-length NOX4 in ischemic cardiomyopathy samples and confirmed presence of shorter isoforms both in control and failing samples with disease-associated expression pattern. We describe here for the first time that NOX4 undergoes extensive alternative splicing in human hearts which gives rise to the expression of different enzyme isoforms. The full length NOX4 is significantly upregulated in ischemic cardiomyopathy suggesting a role for NOX4 in ROS production during heart failure.

## Introduction

In spite of an overall decrease in coronary artery disease-related mortality, the number of patients suffering from heart failure is increasing steeply in aging societies (Rich, 2001; Bui et al., 2011). Aging has a considerable impact on the heart and the vasculature, partially by promoting a prooxidative milieu (Csiszar et al., 2002; Donato et al., 2007; Dai et al., 2012; Martin-Fernandez and Gredilla, 2016). In accordance, increased oxidative stress and subsequent redox imbalance has been implicated in the development and progression of heart failure (Keith et al., 1998; Ungvari et al., 2005). Reactive oxygen species (ROS) production at a basal level may induce profound adaptive changes in intracellular pathways, however, higher concentrations of ROS induces tissue damage. ROS are derived from several intracellular sources, including mitochondrial respiratory complexes, NADPH oxidases (NOX), xanthine oxidase, mono-amino oxidases, and uncoupled nitric oxide synthase, among others. Although, the majority of these enzymes generates ROS as a by-product of dysfunctional activity, the only known role of the NOX family is ROS production. This fact makes NOXs intriguing targets for pharmacotherapy, allowing selective targeting of disease-specific ROS production (Altenhofer et al., 2012, 2015). To our present knowledge, the NOX family is composed of five different enzymes (NOX1, NOX2, NOX3, NOX4, and NOX5) and five subunits, known as phox proteins (Leto et al., 2009; Sirokmany et al., 2016). NOX enzymes have been proven to be involved both in experimental models (Li et al., 2002; Byrne et al., 2003) and in humans suffering from advanced heart failure (Heymes et al., 2003). In a landmark paper, Heymes et al. described an overall increase in NOX activity in human failing hearts, however, surprisingly, they found no change in the overall level of expression of oxidase subunits in failing hearts (Heymes et al., 2003), suggesting a novel NOX as a potential source of ROS production.

The NADPH oxidase 4 (NOX4) isoenzyme has been discovered in 2000, in the renal cortex (Geiszt et al., 2000). However, it has been proven later that many other cell types (including cardiomyocytes) also express NOX4 (Byrne et al., 2003; Varga et al., 2013). According to a recent study, mitochondrial NOX4 activity is a critical regulator of fatty acid β-oxidation in macrophages (Moon et al., 2016), an effect that has been also described in cardiomyocytes (Nabeebaccus et al., 2015).

Interestingly, in contrast to the inducible NOX2, NOX4 displays constitutive mRNA expression, which is fine-tuned by delicate epigenetic mechanisms, involving microRNA-dependent posttranscriptional repression (Varga et al., 2013). In addition, NOX4 might have alternative mRNA splice variants (Goyal et al., 2005) that might further complicate understanding the role of NOX4 in normal physiology and in pathological conditions. Accordingly, in the last decade, several conflicting results have been published, giving rise to many controversy on the NOX4 field. So far, both superoxide and hydrogen peroxide have been proposed as a product of NOX4 activity (Shiose et al., 2001; Takac et al., 2011; Nisimoto et al., 2014). Mitochondrial localization of NOX4 is still a question of debate (Hirschhauser et al., 2015), while a recent paper suggest nuclear/perinuclear localization of NOX4 (Matsushima et al., 2013). In addition, NOX4 has been described both as detrimental and protective in mouse models of heart failure (Kuroda et al., 2010; Zhang et al., 2010; Nabeebaccus et al., 2015; Zhao et al., 2015; Matsushima et al., 2016).

Therefore, here we aimed to characterize and assess the expression of NOX4 in the failing human hearts with unbiased transcriptomics methods followed by validations at the protein level. In addition, we put special emphasis to characterize alternative splicing of NOX4 in healthy and failing human hearts.

## Materials and methods

### Study design

All experimental procedures were done in accordance with the ethical standards of the responsible institutional and national committee on human experimentation, adhering to the Helsinki Declaration (1975). Written informed consent was obtained from all patients involved in the study according to the protocol approved by the Local Ethics Committees of the Institute of Cardiology, Warszawa, Poland and Hospital Universitario Puerta de Hierro Majadahonda, Madrid, Spain (IK-NP-0021-24/1426/14, 272-19/12/11). Healthy human hearts were obtained from organ donor patients (CONT, *n* = 5) whose hearts were not used for transplantation due to technical reasons (e.g., donor/recipient incompatibility). The donors did not have any relevant previous cardiological history or any abnormalities in ECG and echocardiography (LV dimensions/contractility within normal ranges). Explanted failing hearts were obtained from patients suffering from end-stage, advanced heart failure of non-ischemic (i.e., dilated cardiomyopathy, DCM, *n* = 5) or ischemic (ICM, *n* = 5) etiology.

### Preparation of tissue samples

Human tissue samples were taken at the time of heart explanation (avoiding scarred, fibrotic, or adipose tissue, endocardium, epicardium, or coronary vessels). The samples were rinsed immediately in saline, blotted dry, frozen in liquid nitrogen and kept at −80°C until further processing.

Rat heart and kidney tissues were harvested from male Wistar rats (250–300 g). The samples were snap frozen and crushed to small pieces in a mortar with pestle in liquid nitrogen.

### RNA sequencing

Whole transcriptome sequencing was performed, and data regarding NOX4 transcript were used and evaluated in the present project. Total RNA was isolated after homogenization of frozen myocardial samples using RNAeasy columns (Qiagen) as previously described (Lopez-Olaneta et al., 2014). RNA integrity was assessed using an Agilent Bioanalyzer and reverse-transcribed. Amplified cDNA (1 μg) was sonicated to an average size of 100–300 bp and used with the TruSeq DNA Sample Preparation v2 Kit (Illumina) to generate index-tagged sequencing libraries. Libraries were applied to Genome Analyzer IIx (Illumina) followed by standard RNA sequencing protocol to generate 80–120M of paired end 75 bp-long reads. Reads were further processed using the CASAVA package (Illumina) and cutadapt software (Extended Experimental Procedure). Resulting reads were mapped to the ensemble human genome v75 and quantified on the transcriptome using RSEM (Li and Dewey, 2011). From RSEM we used the IsoPct information (percentage of the gene expression accounted by each transcript) in each sample to identify isoforms potentially undergoing alternative splicing.

### Long-distance PCR analysis of alternatively spliced mRNA transcripts

Total RNA was isolated from homogenized left ventricle (LV) of the CONT, DCM, or ICM patients with the guanidinium thiocyanate-phenol-chloroform extraction method, as described earlier (Baan et al., 2015). RNA was reverse transcribed with MMLV-Reverse Transcriptase (Invitrogen, USA). For the detection of the alternatively spliced transcript levels of NOX4, long-distance PCR was carried out with a high fidelity Pfu polymerase (G-Biosciences, USA) with cycling conditions set as an initial denaturation step for 5min at 95°C, followed by 40 cycles of 30 s at 95°C for template denaturation, 30 s for annealing phase at 55°C, and 3.5 min at 72°C for extension. Length of the specific PCR products was verified on 1.5% agarose gels stained with GelRed (Biotium, USA). Primer pairs for the long distance amplification of NOX4 were designed to amplify from a conserved region from all splice variants. The primer sequences were the following: forward primer 5′-TGCTGTATAACCAAGGGCCA-3′, reverse primer 5′-GGTCCACAACAGAAAACACCA-3′. The primers were designed by Primer 3 Input (version 0.4.0) software and tested to avoid primer dimer formation, unspecific amplification and self-priming formation.

### Western blot analysis of NOX4

In order to investigate whether NOX4 expression is altered at the protein level in the failing human heart, western blot analysis was performed. Frozen tissue samples from left ventricle (LV) and right ventricle (RV) as well as from inter-ventricular septum (IVS) were homogenized in NP-40 lysis buffer (150mM NaCl, 50mM Tris, 1% NP-40) for NOX4 or with RIPA buffer for ERK with a tissue to homogenization buffer ratio of 1:4 containing protease and phosphatase inhibitors (AEBSF, Aprotinin, Bestatin, E-64, Leupeptin, Pepstatin A, sodium orthovanadate and sodium fluoride) (Sigma-Aldrich, St. Louis, MO, USA). Protein concentrations were assessed by the bicinchoninic acid method using the provided bovine serum albumin as standard (Pierce Biotechnologies, USA). Equal amounts of protein were loaded from each sample onto 10% SDS-polyacrylamide gels. For optimal results, in case of NOX4 blots, samples were mixed with Laemmli sample buffer without using reducing agents or sample boiling. After separation by electrophoresis, proteins were transferred (wet transfer, 2.5 h) onto nitrocellulose membrane (Amersham Biosciences, Piscataway, USA). Transfer was controlled by Ponceau S staining. The membrane was blocked with 5% non-fat dry milk in 0.1% TBS-T overnight at 4°C. After the blocking step, the membrane was incubated with a rabbit polyclonal primary antibody (dissolved in 1% non-fat dry milk in 0.1% TBS-T, 1:1,000 dilution) against NOX4 (NB110-58851, Novus Biologicals, United Kingdom)—reported to specifically recognize NOX4 protein (Basuroy et al., 2009; Maalouf et al., 2012; Siuda et al., 2012)—, or by a mouse monoclonal antibody recognizing pERK1/2 or total ERK (9106 and 9107 Cell Signaling technology, Danvers, MA, USA) for 2 h at room temperature (NOX4) or overnight at 4°C (ERK), followed by washing with 0.1% TBS-T (3 times for 10 min). After washing, the membrane was incubated with a secondary antibody (horseradish peroxidase-conjugated affinity purified goat anti-rabbit, 1:5,000 dilution, Dako, Denmark; horseradish peroxidase-conjugated affinity purified horse anti-mouse, 1:5,000 dilution, Cell Signaling Technology, USA) in 1% non-fat dry milk in 0.1% TBS-T for 1 h at room temperature. Then the membrane was washed again 3 times for 10 min. For detection of the bands, the membrane was incubated with ECL-Plus reagent (Amersham Biosciences, USA) for 5min and then visualized. Band densities were evaluated by using Quantity One software (Bio-Rad Imaging System, USA). Equal loading was assessed by determining and normalizing to GAPDH content of each sample. Briefly, stripped membranes were probed with a primary antibody that recognizes GAPDH (1:10,000 dilution, Cell Signaling Technology, Danvers, MA, USA) for 2 h at room temperature, followed by washing with TBS-T. Then the membrane was incubated with horseradish peroxidase-conjugated affinity purified goat anti-rabbit antibody (1:20,000 dilution) for 1 h at room temperature. The membrane was washed again and band visualization and evaluation of band densities were done as describe above. There was no significant difference in GAPDH between groups.

### Statistical analysis

Statistical analysis was performed by one-way ANOVA using Prism software (GraphPad Software, Inc., San Diego, USA), as appropriate. All data were expressed as means ± S.E.M. For all analyses, a *p* < 0.05 was considered statistically significant.

## Results

### Study patients

A detailed summary of clinical characteristics and medication of study subjects are shown in Table 1. Patients of both genders were enrolled in all groups. The age of patients suffering from ischemic cardiomyopathy differed significantly from both control and dilated cardiomyopathy patients as expected, since ischemic cardiomyopathy leads to end-stage heart failure later and in older population than dilated cardiomyopathy. DCM and ICM patients were in either New York Heart Association (NYHA) class III or IV with no difference in major cardiac functional parameters. All patients were treated with angiotensin-converting enzyme inhibitors, beta-blockers and diuretics, however, aspirin and statins were only used in the treatment regime of ICM. Control subjects received intravenous treatment composed of very low catecholamine infusion (norepinephrine: 0.1–0.2 μg/kg/min, dopamine: 1–2 μg/kg/min). Adequate fluid balance was maintained before heart explanation with intravenous fluids (e.g., hydroxyethyl starch) and desmopressin.

**Table 1:** Clinical characteristics of study population.

	**CON**	**DCM**	**ICM**
Number of samples	5	5	5
Gender (female/male)	3/2	2/3	4/1
Age (year)	29 ± 9	39 ± 10	57 ± 11
NYHA functional class III/IV, *n*	n.a.	0/5	3/2
LVED, mm	n.a.	68 ± 4	71 ± 4
LVSD, mm	n.a.	63 ± 5	61 ± 8
PW, mm	n.a.	9.5 ± 0.5	10 ± 1.5
IVS, mm	n.a.	10 ± 0.7	11 ± 1.5
LVEF, %	n.a.	16 ± 3	23 ± 3

*Values are given in mean ± S.E.M. CON, healthy control individuals; DCM, dilated cardiomyopathy; ICM, ischemic cardiomyopathy; NYHA, New York Heart Association; LVED, left ventricular end-diastolic diameter; LVSD, left ventricular end-systolic diameter; PW, posterior wall thickness; IVS, interventricular septum thickness; LVEF, left ventricular ejection fraction; n.a., not applicable*.

### NOX4 transcript variants are detected in human hearts

**Figure 1:**
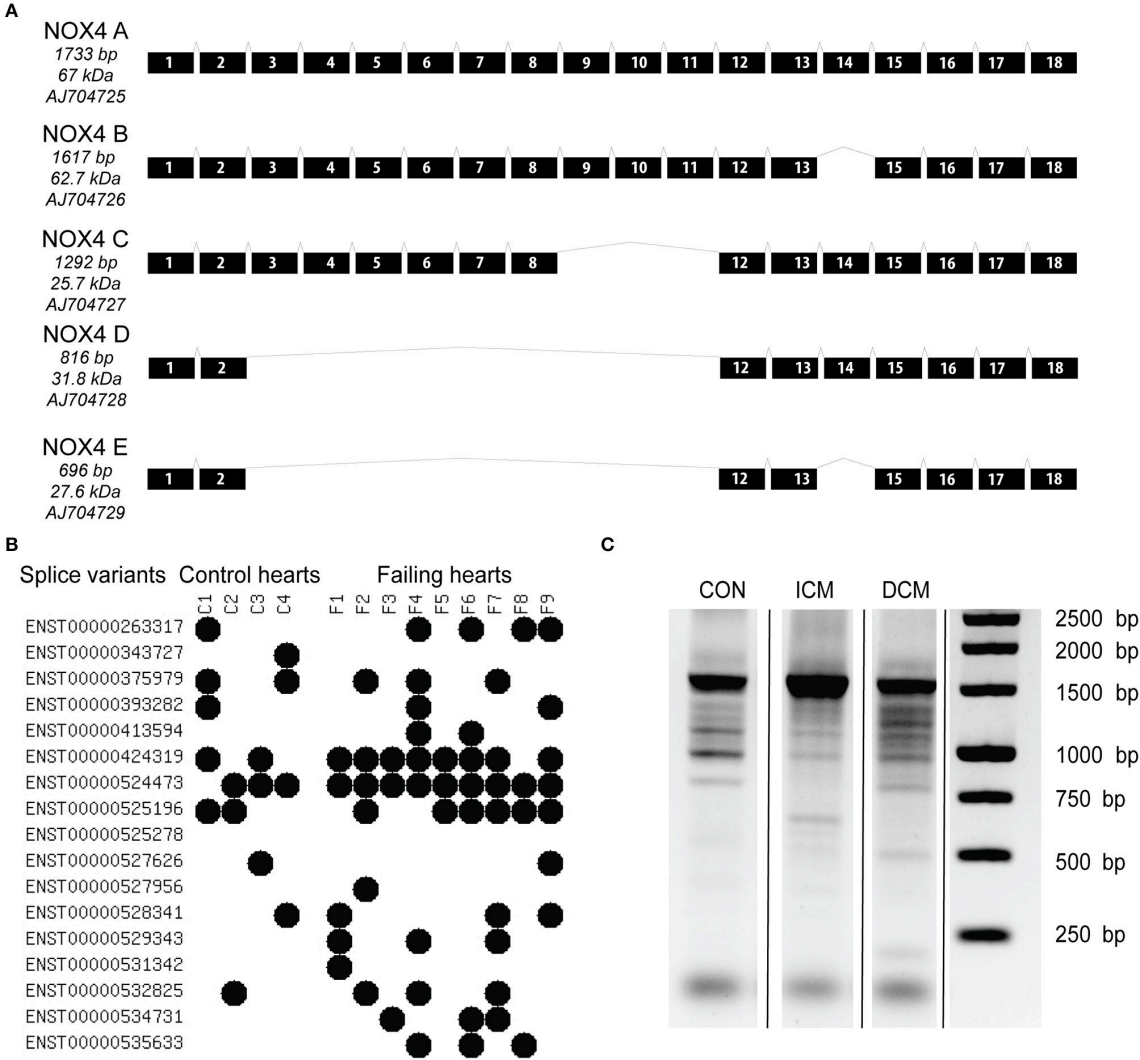
Known transcript variants of the NOX4 messenger RNA as original described in a human endothelial cell line by Goyal et al. (2005) **(A)**. Splice variants detected in control and failing human hearts by RNA sequencing **(B)**. Each variant is annotated to an individual code in the Ensembl database. Black dots represent the presence of the variant in the sample. Long distance PCR analysis to detect NOX4 transcripts **(C)**. CON, control; ICM, ischemic cardiomyopathy; DCM, dilated cardiomyopathy.

The full length NOX4 A consist of 18 different exons, giving rise to the transcription of a 1,733 bp mRNA (Figure 1A). The majority of isoforms lack one or more exons as a result of alternative splicing. With an unbiased RNA sequencing approach, we aimed to characterize the abundance of NOX4 splicing events. (Figure 1B) shows the detection of splice variants (based on the Ensembl database) as detected by RNA sequencing in control and failing human hearts. Currently there are 17 NOX4-related entries, out of them 16 were detected in control and failing human samples. Two sequences are only retained introns (ENST0000524473 and ENST0000525278), and ENST529343 has a premature stop codon potentially leading to non-sense-mediated mRNA decay. However, the retained intron sequence, ENST0000524473 was detectable in almost all samples.

It is also noteworthy that more alternative splicing events were detected in the failing hearts (control average four events, failing average 5.6 events). This was further confirmed by a long distance PCR analysis that is shown on (Figure 1C). By using a universal 5′-3′ end primer pair and a high fidelity enzyme and longer amplification time, we were able the detect several NOX4 related transcripts, showing on one hand that the detected bands are corresponding to the predicted and expected splice variants, while on the other hand, it is also detectable on the agarose gel picture that there is extensive splicing in the failing hearts when compared to the healthy control left ventricular tissue.

### NOX4 protein variants are present in failing human hearts with a spatial and disease-specific distribution

**Figure 2:**
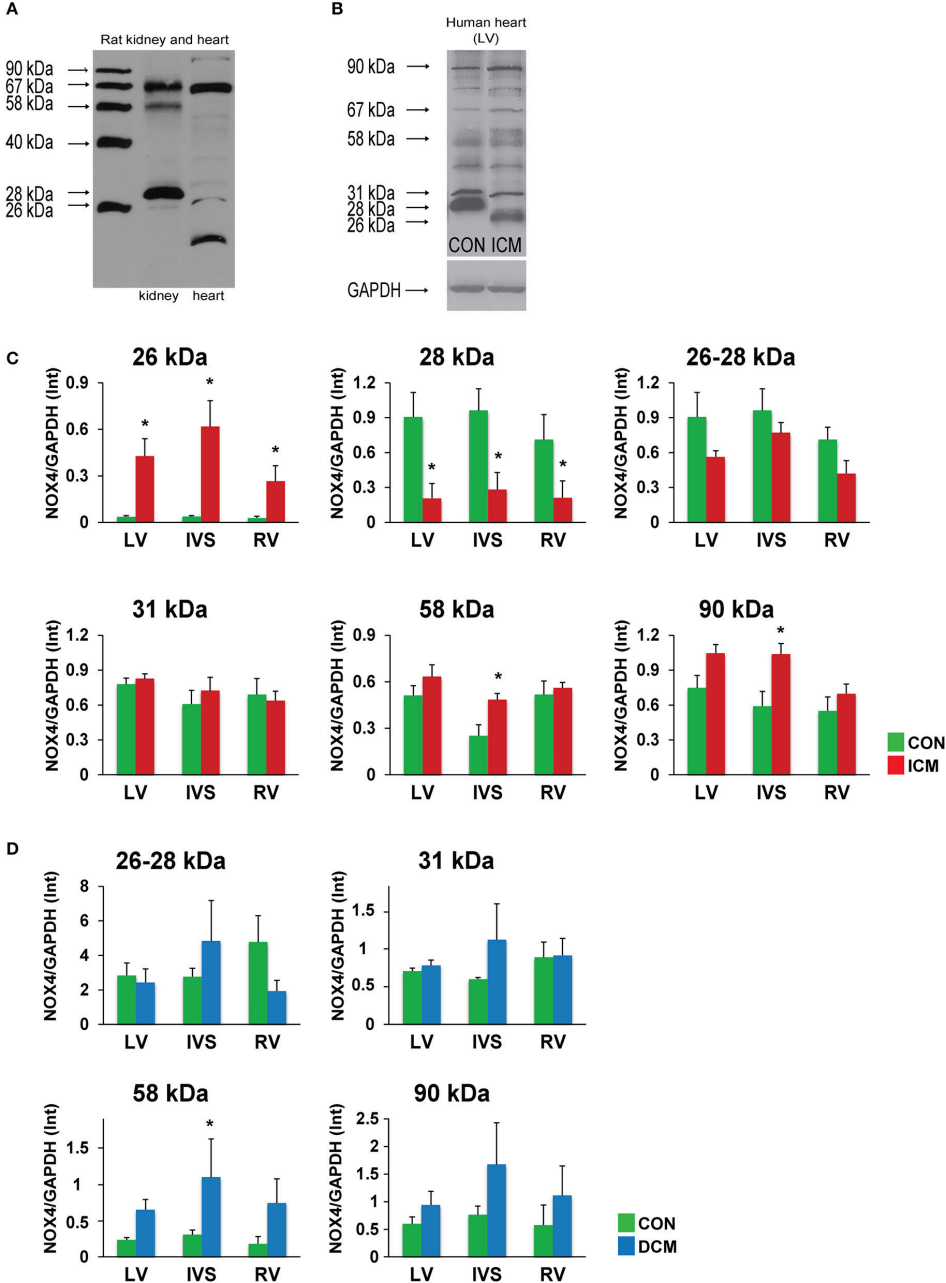
Alternative splicing of NOX4 in rat kidney and heart **(A)**. Alternative splicing of NOX4 in human hearts **(B)**. Quantitative evaluation of spliced NOX4 isoforms in ICM samples **(C)**. Quantitative evaluation of spliced NOX4 isoforms in DCM samples **(D)**. Data are mean ± S.E.M. *n* = 5/group. ^*^p < 0.05. LV, left ventricle; IVS, interventricular septum; RV, right ventricle; ICM, ischemic cardiomyopathy; DCM, dilated cardiomyopathy.

To detect NOX4 variants at protein level, we used a polyclonal antibody recognizing a conserved region of the protein (C-terminal region) present in all transcript variants. During our pilot experiments, we recognized that even in rodent samples (rat kidney and heart) there is some extent of alternative splicing, with a predominant expression of the 67-kDa band in both tissues (Figure 2A).

However, in human hearts and using non-reducing loading conditions this observation became more obvious (Figure 2B, Supplementary Figure 1). Both in healthy control and failing human hearts significant amounts of alternatively spliced forms were detected. In ICM samples, the robust upregulation of the 26-kDa form and downregulation of the 28-kDa form was observed in all regions of the heart. Since the electrophoretic mobility difference between the two forms is small, it is also possible that we detected a posttranslational modification (e.g., deglycosylation—Goyal et al., 2005) of the same isoform. The 58-kDa form showed a significant upregulation in the IVS of the ischemic failing hearts. In addition, we detected a significant increase of the full length NOX4 (in non-reducing conditions the major band is detected at the level of 90 kDa possibly as a complex of a 67-kDa NOX4 with the p22 phox) in the IVS of ischemic failing hearts that was associated with a tendency of upregulation in the left ventricle as well (Figure 2C, Supplementary Figure 1).

In DCM samples, a significant upregulation of the 58-kDa variant was seen, together with a tendency of increased full length 90-kDa complex, similarly to the ICM samples (Figure 2D, Supplementary Figure 2).

To study the link between the observed difference in the expression of the 28-kDa isoform and ERK1/2 phosphorylation (Anilkumar et al., 2013), we performed pERK/ERKWestern blots from left ventricular samples. We found significantly increased phosphorylation of ERK1/2 in the ICM samples as compared to CON samples, whereas there was an increase both in phosphor-ERK and total-ERK levels when normalized to GAPDH in DCM samples. These suggest activation of hypertrophic ERK signaling independently from changes in NOX4D expression in these samples (Supplementary Figure 3).

## Discussion

We described here for the first time that the NOX4 gene undergoes alternative splicing in the human heart resulting in at least three different protein isoforms. The full length NOX4 is significantly upregulated in heart failure, while a smaller 28-kDa isoform shows downregulation in ischemic failing hearts.

Alternative splicing is a critical process in RNA maturation ensuring expression of functionally diverse proteins from individual genes. Frequency of splicing events is estimated to be really high, according to Pan and colleagues more than 85% of the multi-exon genes contain at least one alternative splicing event (Pan et al., 2008). Since alternative splicing is usually tissue specific, and in many cases changes in alternative splicing occur in a disease-specific manner during progression of the disease, detection of splicing events could serve as a disease-specific or disease stage-specific biomarker. In addition, regulation of alternative splicing might affect disease outcome, pointing to the theranostic potential of splicing events (Le et al., 2015). Although, gene expression patterns in cardiac diseases have been extensively studied over the last years, our overall knowledge in terms of splicing events and the resulting protein isoforms and their association with disease states is still very limited. The variability in splicing may alter protein structure, thereby influencing subcellular protein localization, and the overall function of the particular protein. In the myocardium, alternative splicing of sarcomeric genes, cardiac ion channels, and cell signaling proteins have been reported so far, leading to the development of cardiomyopathies and arrhythmias (Gao et al., 2011; Guo et al., 2012; Lara-Pezzi et al., 2013; Maatz et al., 2014). Among NOX family members, it is known that both NOX1 (Arakawa et al., 2006) and NOX2 (Kuhns et al., 2010; Harrison et al., 2012) enzymes undergo alternative splicing. Interestingly, in lymphocytes of patients suffering from chronic granulomatosus disease due to mutations in the CYBB gene (i.e., gp91 phox) IFN-γ is capable of partially correcting mRNA processing defects and improves splicing efficiency (Condino-Neto and Newburger, 2000). To date NOX4 transcripts variants have been reported only in cell lines, including alveolar epithelial cells (Goyal et al., 2005), vascular smooth muscle cells, endothelial cells, fibroblasts, and cardiomyocytes (Anilkumar et al., 2013). Nevertheless, the functional role of the transcripts is still largely unknown. Anilkumar et al. have reported that the 28-kDa splice variant (aka. NOX4D) is predominantly expressed in the nucleus, and produces ROS that can activate kinase signaling cascades, such as MAPK, and ROS-dependent extracellular-signal-regulated kinases (ERK1/2) (Anilkumar et al., 2013), that may potentially contribute to nuclear redox homeostasis (Hansen et al., 2006). These observations are interesting in light of our present results, since we observed a marked switch in the expression/posttranslational modification of the 28-kDa isoform, showing lower expression levels in the hearts of ischemic cardiomyopathy patients. In our samples, however, we found increased ERK phosphorylation both in ICM and DCM samples, suggesting a potentially NOX4D-independent activation of hypertrophic signaling during heart failure (Rose et al., 2010; Yeh et al., 2010).

Given the controversies in the NOX4 field, differences in splicing events might also contribute to the different phenotypes seen in the NOX4 knockout animals. So far four different knock-out models have been published with deletions of different exons (exons 1/2, exon 4, exon 9, or exons 14/15; for review please see: Altenhofer et al., 2012). Therefore, it is possible that, in a tissue specific manner, deletion of exon 4 or exon 9 may result in the expression of NOX4 variants (NOX4D and E) leading to residual NOX4 activity that complicates the interpretation of the results seen in knock-out mice studies.

From a drug developmental point of view, alternative splicing might be an important factor to consider as drug candidates may have different effects on the spliced variants (e.g., a splice event might underlie drug resistance de Necochea-Campion et al., 2016). Therefore, NOX4 inhibitors currently under clinical testing (e.g., GKT137831 from GenKyoTex S.A., VAS2870 from Vasopharm GmbH, GLX351322 from Glucox Biotech AB—see for review: Altenhofer et al., 2015) may also affect the activity of splice variants differently that may in turn influence efficacy and safety.

## Limitations

A limitation of the present observational study is that our conclusions are based on descriptive data from a limited set of human samples. Due to the significant differences in the age of the ICM patients and the healthy controls, we cannot rule out the effect of age and the presence of different cardiovascular comorbidities (Ferdinandy et al., 2007, 2014) on the observed differences. Although mRNA and protein data clearly implicate the presence of the alternative splice variants of NOX4, we have not provided direct evidence by sequencing the proteins after immunoprecipitation with the antibody used in the present study.

## Conclusions

In summary, our present study provides the first description that the NOX4 mRNA undergoes alternative splicing in the human heart, resulting in at least three different protein isoforms. The full length NOX4 is significantly upregulated due to heart failure that might contribute to ROS production in the failing hearts, while a smaller 28-kDa isoform shows downregulation in ischemic failing hearts possibly having important roles in redox signaling of subcellular compartments. Disease specific expression pattern of NOX4 isoforms may provide new diagnostic and therapeutic targets in heart failure, and disease-specific splicing events might represent a new factor to consider when developing NOX4-modulator drugs.

## Author contributions

ZVV, MP, JAB, TB, GK, GJB, ZG, and LM performed *in vitro* experiments. ZVV analyzed data, drafted figures, and the manuscript. PL, MK, and PG-P provided patient materials and clinical data. FS-C performed bioinformatic analysis. ZVV, LM, TC, EL-P, PP, and PF conceptualized the project, provided necessary materials, and wrote the manuscript.

## Conflict of interest statement

The authors declare that the research was conducted in the absence of any commercial or financial relationships that could be construed as a potential conflict of interest.

## Supplementary material

Supplementary Figure 1 Original Western blots for NOX4 **(A)**, GAPDH **(B)**, and total protein staining by Ponceau S stain **(C)** in left ventricular (LV), right ventricular (RV), and interventricular septal regions (IVS) of ischemic cardiomyopathy patients (ICM).

Supplementary Figure 2 Original Western blots for NOX4 **(A)**, GAPDH **(B)**, and total protein staining by Ponceau S stain **(C)** in left ventricular (LV), right ventricular (RV), and interventricular septal regions (IVS) of dilated cardiomyopathy patients (DCM).

Supplementary Figure 3 Original Western blots for phosphorylated ERK1/2, total ERK1/2, GAPDH and total protein staining by Ponceau S stain in left ventricular (LV) samples of ischemic (ICM—**A**) or dilated cardiomyopathy patients (DCM—**B**), respectively. Data are mean ± S.E.M. *n* = 5/group. ^*^*p* < 0.05.
