# Supplementary figures and images for: Alternative Splicing of NOX4 in the Failing Human Heart
Source: Front Physiol. 2017 Nov 22;8:935. doi: 10.3389/fphys.2017.00935 (PMC5698687; doi:10.3389/fphys.2017.00935)

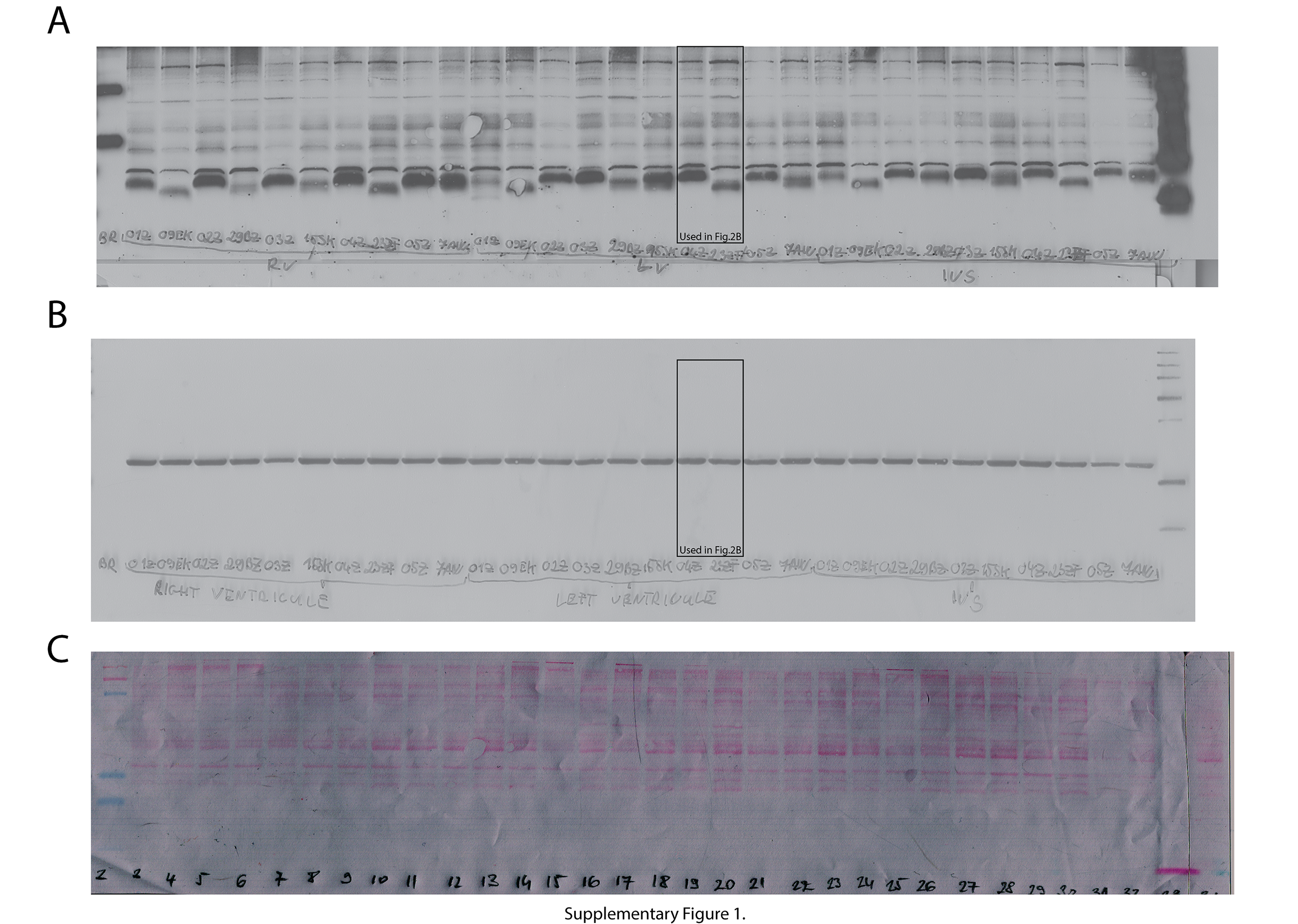

Supplement: Supplementary Figure 1 — Original Western blots for NOX4 (A), GAPDH (B), and total protein staining by Ponceau S stain (C) in left ventricular (LV), right ventricular (RV), and interventricular septal regions (IVS) of ischemic cardiomyopathy patients (ICM). [file Image1.TIF]

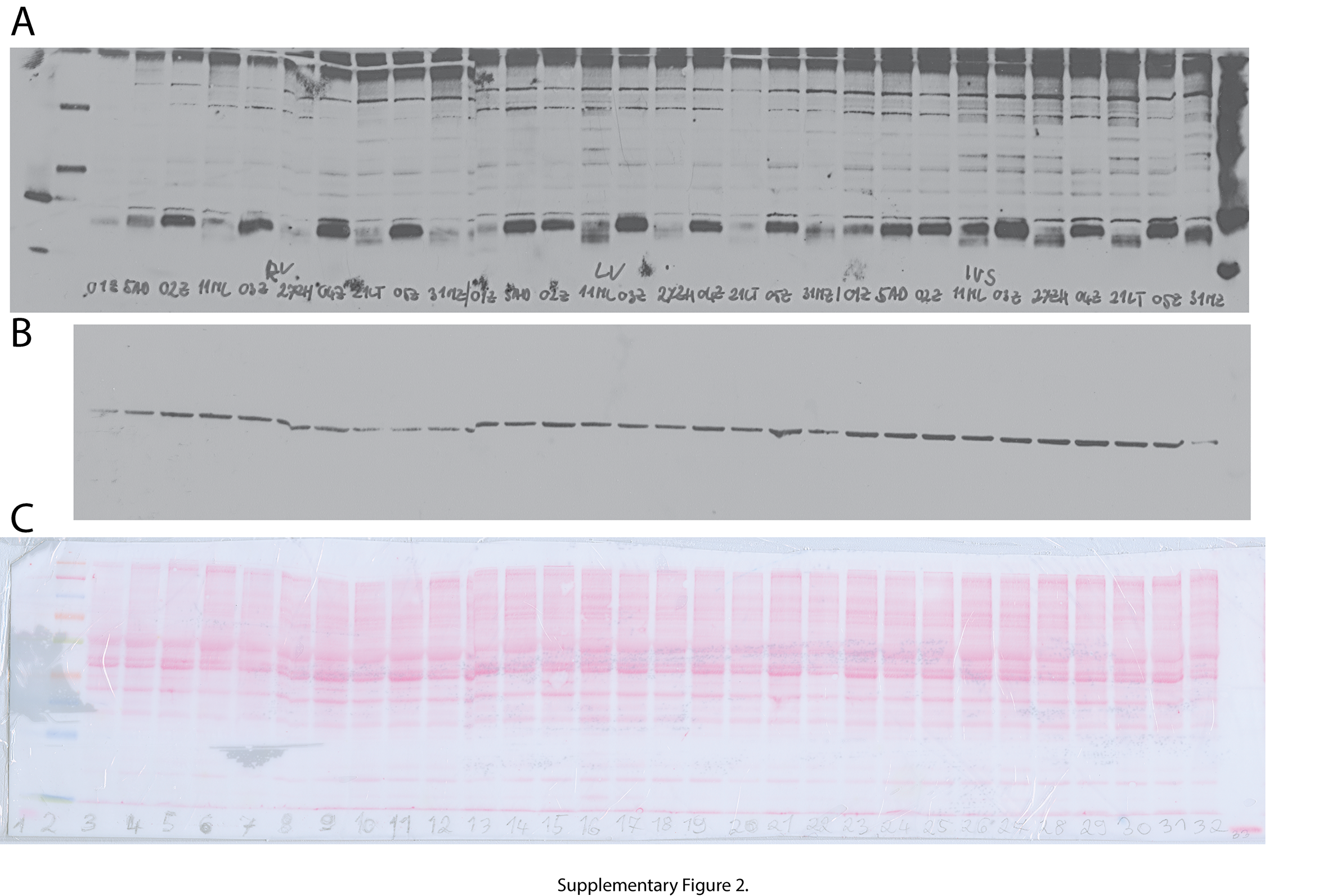

Supplement: Supplementary Figure 2 — Original Western blots for NOX4 (A), GAPDH (B), and total protein staining by Ponceau S stain (C) in left ventricular (LV), right ventricular (RV), and interventricular septal regions (IVS) of dilated cardiomyopathy patients (DCM). [file Image2.TIF]

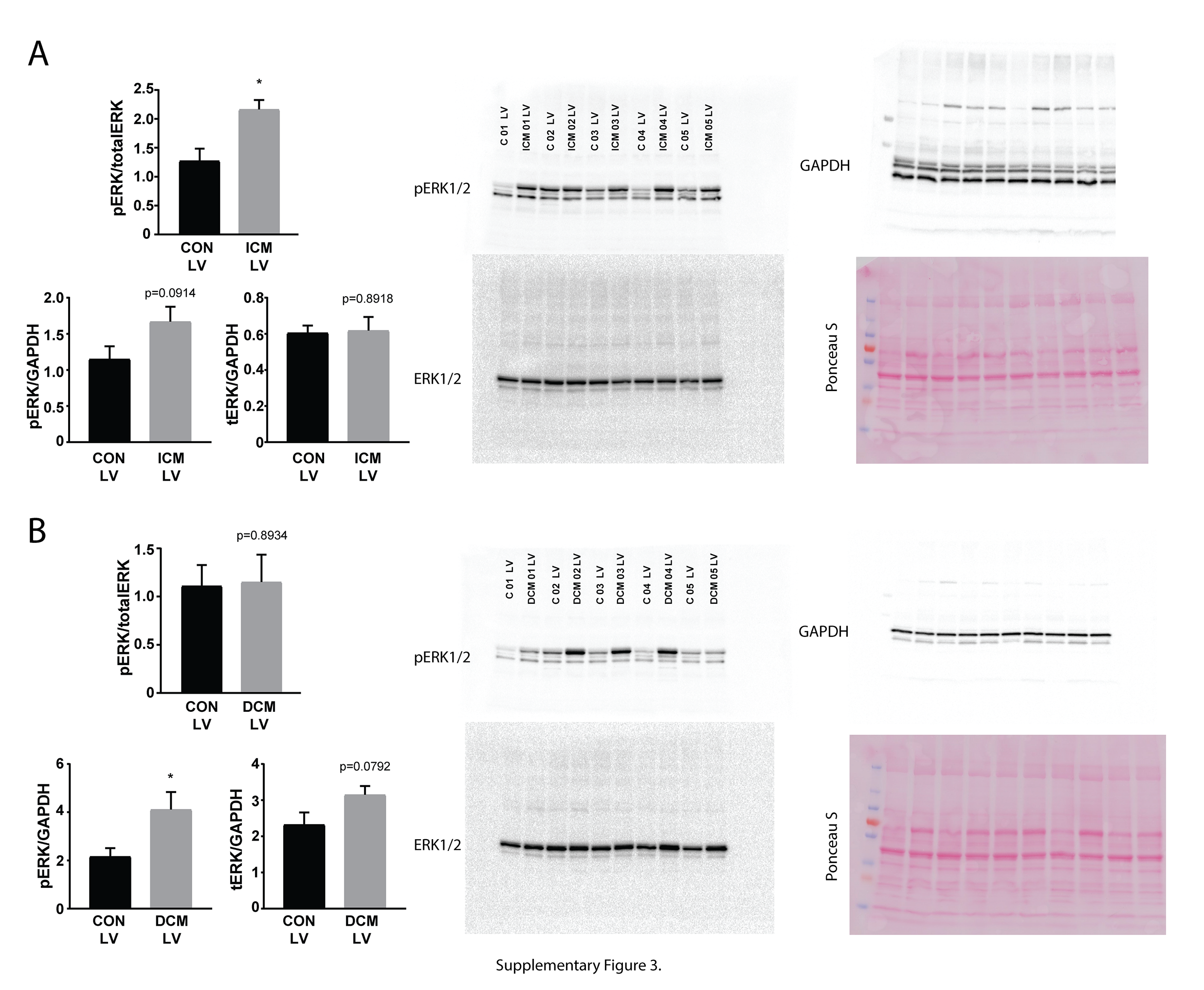

Supplement: Supplementary Figure 3 — Original Western blots for phosphorylated ERK1/2, total ERK1/2, GAPDH and total protein staining by Ponceau S stain in left ventricular (LV) samples of ischemic (ICM—A) or dilated cardiomyopathy patients (DCM—B), respectively. Data are mean ± S.E.M. n = 5/group. *p < 0.05. [file Image3.TIF]
